# Barriers and Facilitators to Continuity of Care in Rural Geriatric Rehabilitation: A Qualitative Study in Malaysia

**DOI:** 10.21315/mjms-03-2026-195

**Published:** 2026-08-31

**Authors:** Jia Yin Teh, Yasothai Sahadeevan, Chung Khian Yew, Ji Chen Lim

**Affiliations:** 1 Department of Rehabilitation Medicine, Hospital Seberang Jaya, 13700 Perai, Pulau Pinang, Malaysia; 2 Department of Rehabilitation Medicine, Faculty of Medicine, Universiti Malaya, 50603 Kuala Lumpur, Malaysia; 3 Department of Rehabilitation Medicine, Hospital Sultanah Bahiyah, 05460 Alor Setar, Kedah, Malaysia; 4 Independent Researcher, Malaysia

**Keywords:** older adults, geriatric rehabilitation, continuity of care, rural health services, qualitative research, telehealth

## Abstract

**Background:**

Older adults in rural areas often face barriers to rehabilitation that affect continuity of care. This study explored barriers to and facilitators of geriatric rehabilitation in rural Malaysia.

**Methods:**

A qualitative phenomenological study involved 30 older adults aged ≥ 60 years who received rehabilitation services between 2018 and 2024, with caregiver proxies participating when necessary. Semi-structured telephone interviews were conducted in Malay, Chinese, or English and were audio-recorded. The recordings were transcribed and translated into English using an automated speech recognition model (OpenAI Whisper). Transcripts were analysed using an AI-assisted thematic analysis framework and classified into eight predefined domains based on sentence-embedding similarity with human review. Segments were manually coded as barriers or facilitators, and scored for sentiment using a rule-based tool (NLTK VADER). Inter-coder reliability was assessed using Cohen’s kappa.

**Results:**

Distance to rehabilitation services and limited transportation were the main barriers to the continuity of care. Financial costs related to travel, treatment, and assistive devices further limited attendance, and some participants reported service constraints. Strong family and community support facilitated rehabilitation engagement. Telehealth was frequently suggested as a means of providing rehabilitation guidance and maintaining continuity of care.

**Conclusion:**

Continuity of geriatric rehabilitation in rural Malaysia is constrained by long travel distances and transportation barriers. Strengthening family support and incorporating simple telehealth approaches may improve continuity of care for older adults in rural communities.

## Introduction

Geriatric rehabilitation is essential for restoring function and quality of life. However, rural hospitals face distinct constraints such as long travel distances, limited transport, workforce shortages, and financial barriers that disrupt continuity of care (1–3). This study was conducted at Hospital Sungai Bakap, which serves a predominantly rural community, making it a suitable setting to examine how these challenges affect rehabilitation among older adults.

Geriatric rehabilitation integrates physical, occupational, and social interventions to sustain independence among older adults. However, rural environments magnify systemic constraints through intersecting barriers related to access, workforce capacity, and socioeconomic conditions (1, 3, 4). Long travel distances, limited transportation, and sparse local services disrupt the continuity of care, whereas shortages of trained professionals reduce service quality (5, 6). Similarly, family and community support, multidisciplinary transitional care, and telehealth can help sustain rehabilitation when access to formal services is limited (7–10).

Cultural and behavioural factors, low health literacy, and environmental and infrastructural constraints further shape help-seeking and engagement with rehabilitation, although targeted education and cost-effective models such as comprehensive geriatric care and telehealth show promise in rural settings (11–22).

Despite growing international evidence on rural rehabilitation, little is known about how older adults in rural Malaysia experience continuity of care, and few studies have examined the barriers and facilitators within this context. Therefore, this study aimed to explore the barriers to and facilitators of continuity of care in geriatric rehabilitation among older adults in rural Malaysia using a qualitative phenomenological approach.

## Methods

### Study Design

This study used a phenomenology-guided qualitative approach to understand the experiences of older adults undergoing rehabilitation in a rural setting. This approach is useful because it focuses on individuals’ lived experiences and is therefore well suited to capturing how older adults perceive the factors shaping their continuity of care. The study was conducted at Hospital Sungai Bakap, which serves a predominantly rural population and provides an appropriate setting to examine how geographic, infrastructural, and social factors intersect to influence rehabilitation experiences.

### Participants and Sampling

Participants were adults aged 60 years and older who received geriatric rehabilitation services at Hospital Sungai Bakap between 2018 and 2024. Purposive sampling was used to ensure diversity in age, sex, ethnicity, medical condition, and duration of rehabilitation. When an older adult was unable to participate directly due to communication or cognitive limitations, a primary caregiver or close family member was invited to participate as a proxy respondent. Thirty participants were interviewed (Table 1). Recruitment continued until thematic saturation was reached. A small number of individuals who were approached either declined or could not be contacted and were not replaced, consistent with the qualitative design.

### Data Collection

Data were collected through semi-structured interviews conducted remotely via telephone to enhance accessibility for participants living in rural areas. Members of the research team trained in qualitative interviews conducted the interviews. An interview guide was developed based on the study objectives and existing literature, covering experiences of accessing rehabilitation services, perceived barriers and facilitators, family and community support, and views on continuity of care. The guide used open-ended questions with follow-up prompts to encourage participants to share more details (for example, “Can you tell me more about that?” and “What made it easier or more difficult for you?”). The interviews were conducted in the participant’s preferred languages (Malay, Chinese, or English). Each interview lasted approximately 30 min to 60 min and was audio-recorded with consent. Field notes were taken during and immediately after the interviews. The interviews continued until thematic saturation was reached, defined as the point at which no new substantive themes emerged from the data.

### Data Preparation and Analysis

The interview recordings were transcribed verbatim and, where required, translated into English using an automated speech recognition model (OpenAI Whisper, large-v3). Transcripts were kept in their original language as verbatim records and as the source of all reported quotations, whereas English versions were used for the computational analysis steps. A member of the research team reviewed a sample of transcripts against audio recordings to verify transcription accuracy, and identifying information was manually removed before analysis.

Transcripts were analysed using an AI-assisted thematic analysis framework based on eight predefined themes: access to care, service quality, socioeconomic circumstances, cultural and behavioural influences, environmental and infrastructural factors, reflections on rehabilitation outcomes, family and community support, and telehealth. Each text segment was assigned to the closest matching domain using sentence-embedding similarity (sentence-transformers, all-mpnet-base-v2), and segments with low matching confidence were reviewed and assigned by a researcher. Two researchers separately coded a subset of the transcripts. They then compared their coding, assessed agreement using Cohen’s kappa, and discussed any differences until consensus was reached.

For each coded segment, the researcher recorded whether it represented a barrier or facilitator, and sentiment polarity was scored automatically using a rule-based sentiment tool (NLTK VADER) applied to the English text. The number of segments, proportion expressed as barriers or facilitators, and mean sentiment scores for each domain were aggregated to summarise the findings. The overall workflow of the analysis is shown in Figure 1. Quantitative summaries are presented in Table 2 and Figures 2 and 3, and illustrative quotations are reported in English, translated where necessary from the participants’ original language. All the computational steps were implemented in Python.

### Ethical Approval and Consent to Participate

This study was approved by the Medical Research and Ethics Committee, Ministry of Health Malaysia (NMRR ID-25-00758-ISN; Research ID RSCH ID-24-06744-U2P), and conducted in accordance with the Declaration of Helsinki and Malaysian Good Clinical Practice Guidelines. Written or verbal informed consent was obtained from all participants, and proxy consent was obtained from caregivers when necessary. Participation was voluntary, and respondents could withdraw from the study or skip any questions at any time. As the study involved only minimal-risk interviews, counselling support was available if emotional discomfort occurred. All recordings and transcripts were pseudonymised and stored on encrypted, password-protected institutional servers accessible only to the research team. In line with Malaysian data protection laws, identifiable data were retained for two years before secure deletion. The investigators report no conflicts of interest, and the study was sponsored by Hospital Sungai Bakap.

## Results

### Participant Characteristics

Thirty participants were included in the study, comprising older adults aged 60 years and older and, where appropriate, their caregivers. Participants represented a range of ages, genders, ethnic backgrounds, and medical conditions, with neurological conditions, such as stroke, being the most common reason for rehabilitation. Most participants were female and aged 60 to 79 years. Demographic details are presented in Table 1.

### Overview of Key Themes

The interview analysis identified several interrelated themes reflecting barriers to and facilitators of geriatric rehabilitation in a rural setting. These themes included access to care, service quality, socioeconomic factors, cultural and behavioural influences, environmental and infrastructural constraints, family and community support, telehealth experiences, and reflections on rehabilitation outcomes. Participants more frequently described barriers related to access to rehabilitation services and maintaining continuity of care. In contrast, family and community support and telehealth were commonly discussed as factors that helped older adults remain engaged in rehabilitation. A summary of the themes and their distribution is shown in Table 2 and Figures 2 and 3.

### Access-Related Barriers

Difficulties related to access emerged as the main barrier to rehabilitation. Many participants described long travel distances to the hospital, limited transportation options, and physical effort required to attend appointments as discouraging factors. For some participants, the time and fatigue associated with travel reduced the frequency of attendance and led to missed sessions. These challenges were often compounded by mobility limitations and reliance on family members for transport, making regular attendance difficult, particularly for those with limited social support.

“From our side, it usually takes approximately 45 minutes to one hour to get there.”

“It is quite far.”

“It is far from home.”

### Socioeconomic Constraints

Participants frequently highlighted the financial challenges associated with rehabilitation, including out-of-pocket costs for transport, therapy sessions, medications, and assistive equipment. These expenses were described as burdensome, particularly for older adults with limited incomes. Financial concerns could influence decisions regarding whether to continue rehabilitation or seek additional services outside the hospital.

“It is not provided free of charge.”

“Most of the time, we have to pay for it ourselves.”

“I pay by myself.”

### Service Quality and Communication

Experiences of service quality varied across participants. While some participants described constraints related to staffing and time, many valued clear explanations, caregiver education, and guidance on home-based exercises. The participants reported that practical instructions and reassurance from healthcare providers increased their confidence in continuing rehabilitation after discharge.

“There is… they sort of teach how to do physiotherapy at home… there is.”

“They taught me, I remember there was.”

“They did teach you all.”

### Family and Community Support

Family and community support emerged as key facilitators of rehabilitation continuity. The participants described how family members assisted with transport, supervised exercises, and provided emotional encouragement. In some cases, neighbours or community members also offered support. Informal support networks played a crucial role in helping older adults adhere to home-based rehabilitation, particularly when access to formal services is limited.

### Telehealth as a Supportive Strategy

Telehealth was commonly viewed as a helpful and acceptable option for follow-ups and exercise guidance. Participants expressed openness to phone calls and video consultations, particularly when in-person visits were difficult. Telehealth was perceived as a convenient way to maintain contact with healthcare providers and receive ongoing guidance, although the participants generally viewed it as a supplement rather than a replacement for face-to-face care.

“The doctor conducts video calls and teaches through the phone; it is better than going to the hospital.”

“Yes, this is very good, using video to teach how to perform physiotherapy.”

“In the future, physiotherapy could be conducted via video calls, teaching basic exercises.”

### Interrelationships Between Themes

Based on the participants’ accounts, barriers and facilitators of rehabilitation were highly interconnected rather than experienced in isolation. Difficulties in access to care were often described alongside challenges related to service quality and financial constraints, with long travel distances frequently amplifying the time, costs, and fatigue associated with attendance. These overlapping challenges contributed to difficulties in continuity of care.

Similarly, family and community support were closely linked to participants’ ability to navigate access barriers. Family members often mitigated transport difficulties and financial strain, while also supporting adherence to home-based rehabilitation. Telehealth experiences were commonly discussed in relation to access and service delivery, particularly as a means of maintaining contact with healthcare providers when in-person visits were difficult. These interrelationships highlight how structural, social, and service-related factors collectively shape the rehabilitation experiences of older adults in rural settings, highlighting the need for integrated and context-sensitive approaches to geriatric rehabilitation.

## Discussion

### Central Barriers to Continuity of Geriatric Rehabilitation in Rural Settings

The continuity of geriatric rehabilitation in rural settings is shaped by a combination of structural, service-related, and social factors. Participants in this study commonly described barriers to accessing care, including long travel distances, limited transportation options, and financial constraints, which made it difficult to attend rehabilitation sessions consistently. These experiences are consistent with those reported in previous Malaysian and international studies, highlighting how geographic isolation and transport limitations disrupt rehabilitation participation among older adults living in rural areas (5, 6).

In addition, workforce shortages and limited rehabilitation resources were perceived to affect the quality and continuity of care. Participants reported that limited therapist availability and insufficient time for interaction reduced opportunities for feedback, exercise progression, and reassurance. Over time, these constraints may contribute to reduced engagement with rehabilitation and slower functional recovery, particularly among older adults who require ongoing encouragement and support (3, 20). Together, these findings illustrate how structural barriers intersect with age-related vulnerabilities to shape rehabilitation experiences in rural settings.

### Family and Community Support as a Key Facilitator

Despite these challenges, family and community support emerged as important factors that enabled older adults to continue their rehabilitation. Participants frequently described their reliance on family members for transportation, financial assistance, supervision of home exercises, and emotional encouragement. In some cases, neighbours or community members provided informal support when family members were unavailable. Such support networks are particularly important in rural settings, where access to formal healthcare services may be limited (23).

Family involvement appeared to play a dual role by addressing both practical needs and emotional well-being. Clear communication from healthcare providers, combined with caregiver education, helped families feel more confident in supporting home-based rehabilitation. These findings align with those of previous studies showing that caregiver engagement and social support can enhance rehabilitation adherence and sustain continuity of care among older adults (11, 17). In Malaysia, where multigenerational households and strong family ties are common, strengthening caregiver involvement may be a culturally appropriate and sustainable strategy.

### Telehealth as a Supportive Strategy for Continuity of Care

Participants frequently discussed telehealth as a helpful and acceptable option for maintaining contact with rehabilitation providers, particularly when in-person attendance was difficult. Older adults described telephone- and video-based follow-ups as convenient and less physically demanding, reducing travel burdens and associated costs. These findings are consistent with studies suggesting that telehealth can support continuity of care and improve access for older adults in rural areas (6, 8, 10).

However, telehealth should be viewed as complementary to rather than a substitute for in-person rehabilitation. Participants in this study were already engaged with rehabilitation services and reported basic familiarity with mobile phone use, which may not reflect the experiences of all older adults in rural settings. Barriers related to digital literacy, internet connectivity, and device access are important considerations (13, 18). When implemented thoughtfully, low-intensity telehealth interventions, such as scheduled check-ins or remote exercise guidance, may help sustain rehabilitation engagement while accounting for these limitations.

### Implications for Practice and Policy

The findings highlight the importance of context-sensitive approaches to geriatric rehabilitation in rural settings. Addressing access barriers through improved transportation support, enhancing communication during discharge planning, and strengthening caregiver education may help mitigate disruptions to rehabilitation continuity. Simultaneously, the modest integration of telehealth into existing services may offer additional support without replacing face-to-face care. From a policy perspective, efforts to improve rural rehabilitation services should consider the interconnected nature of access, service capacity, and social support. Strategies that recognise family caregivers as key partners in care while addressing workforce and infrastructural constraints may contribute to more equitable and sustainable rehabilitation services for older adults in rural communities.

### Limitations

This study had several limitations. The participants were recruited from a single rural public hospital (Hospital Sungai Bakap), which may limit the transferability of the findings to other rural settings with different healthcare resources or community contexts. Data were based on self-reported accounts from older adults and caregivers and may therefore be subject to recall or social desirability bias, although open-ended interviews helped elicit detailed perspectives. Interviews were conducted by telephone, which may have limited observation of non-verbal cues; however, this approach improved accessibility for participants living in remote areas. All participants had engaged with rehabilitation services and may represent a relatively motivated group, potentially underrepresenting older adults who disengaged early or faced greater cognitive, sensory, or digital barriers. Finally, although the analysis was conducted in the original interview languages, translation of selected quotations into English may not have fully captured all cultural nuances.

## Conclusion

In summary, continuity of geriatric rehabilitation in rural Malaysia is constrained primarily by long travel distances, limited transport, financial burdens, and workforce shortages. Strong family and community support and acceptance of simple telehealth approaches emerged as the main facilitators of continued engagement with rehabilitation. These findings highlight the need for practical strategies suited to rural settings, such as improving transport support, strengthening caregiver education, and thoughtfully introducing low-intensity telehealth services. Future studies should evaluate these approaches in larger, more diverse rural populations.

## Figures and Tables

**Figure 1:**
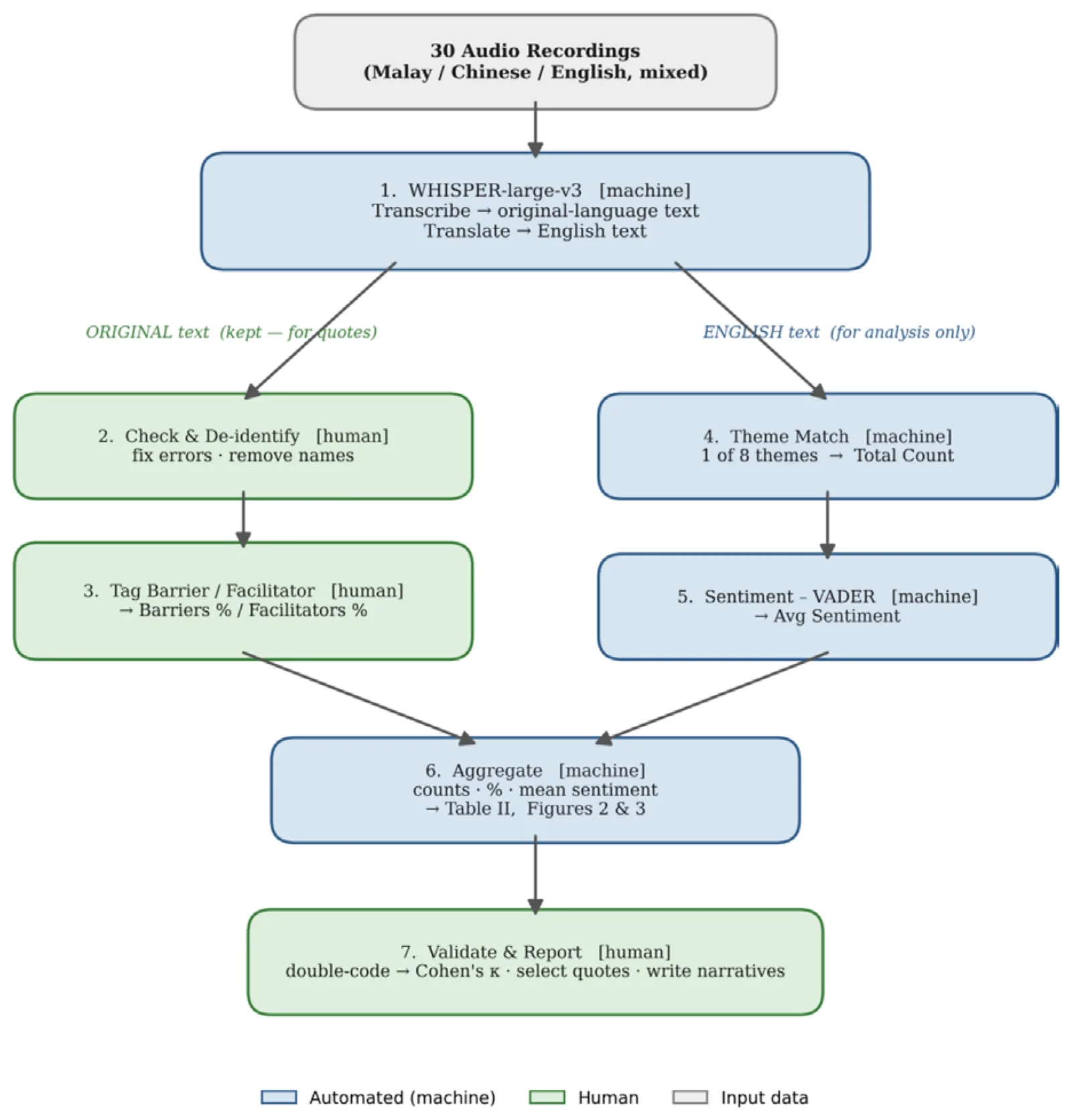
AI-assisted analysis pipeline for transcription, theme classification, and sentiment scoring.

**Figure 2:**
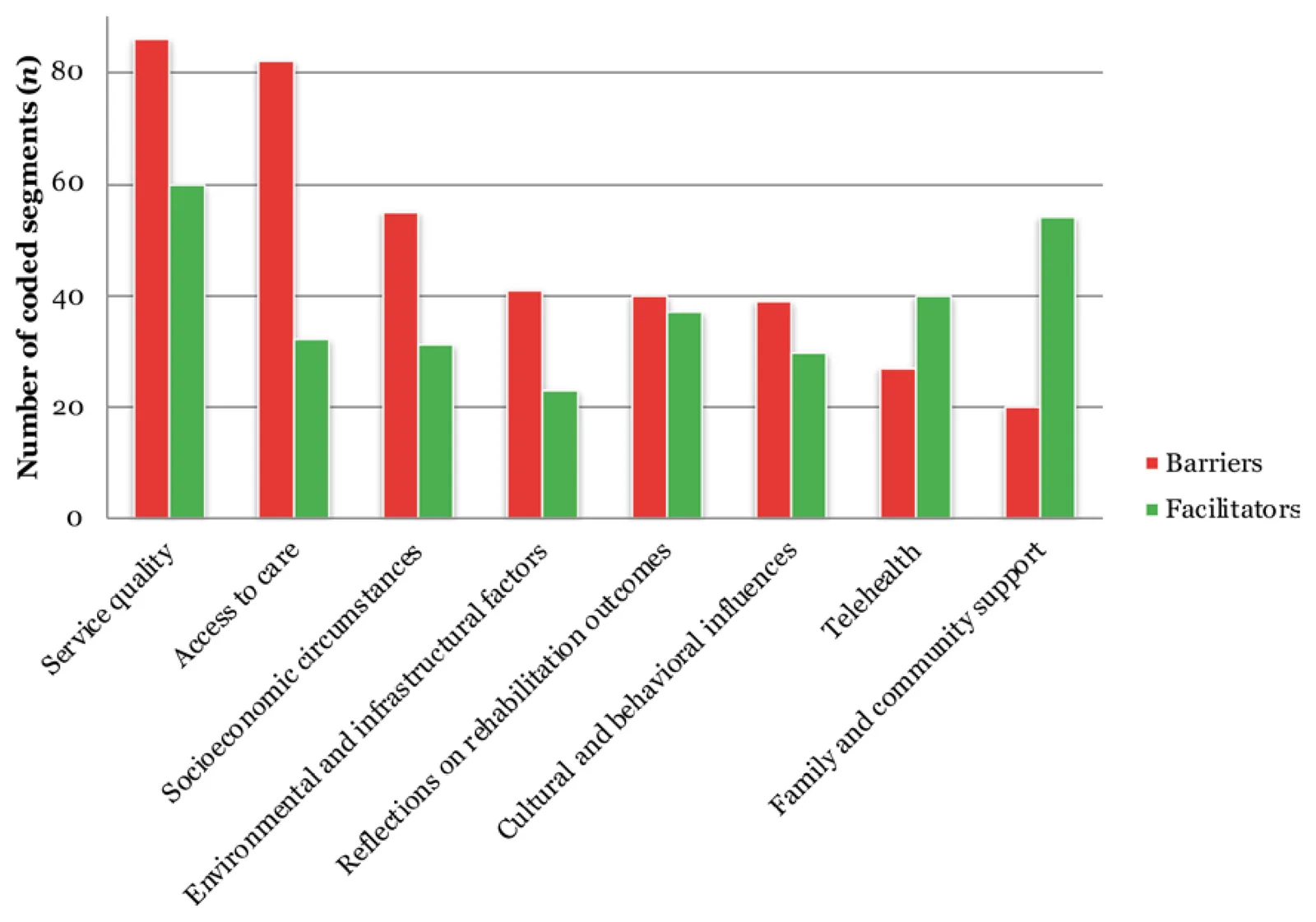
Grouped bar chart of theme prevalence.

**Figure 3:**
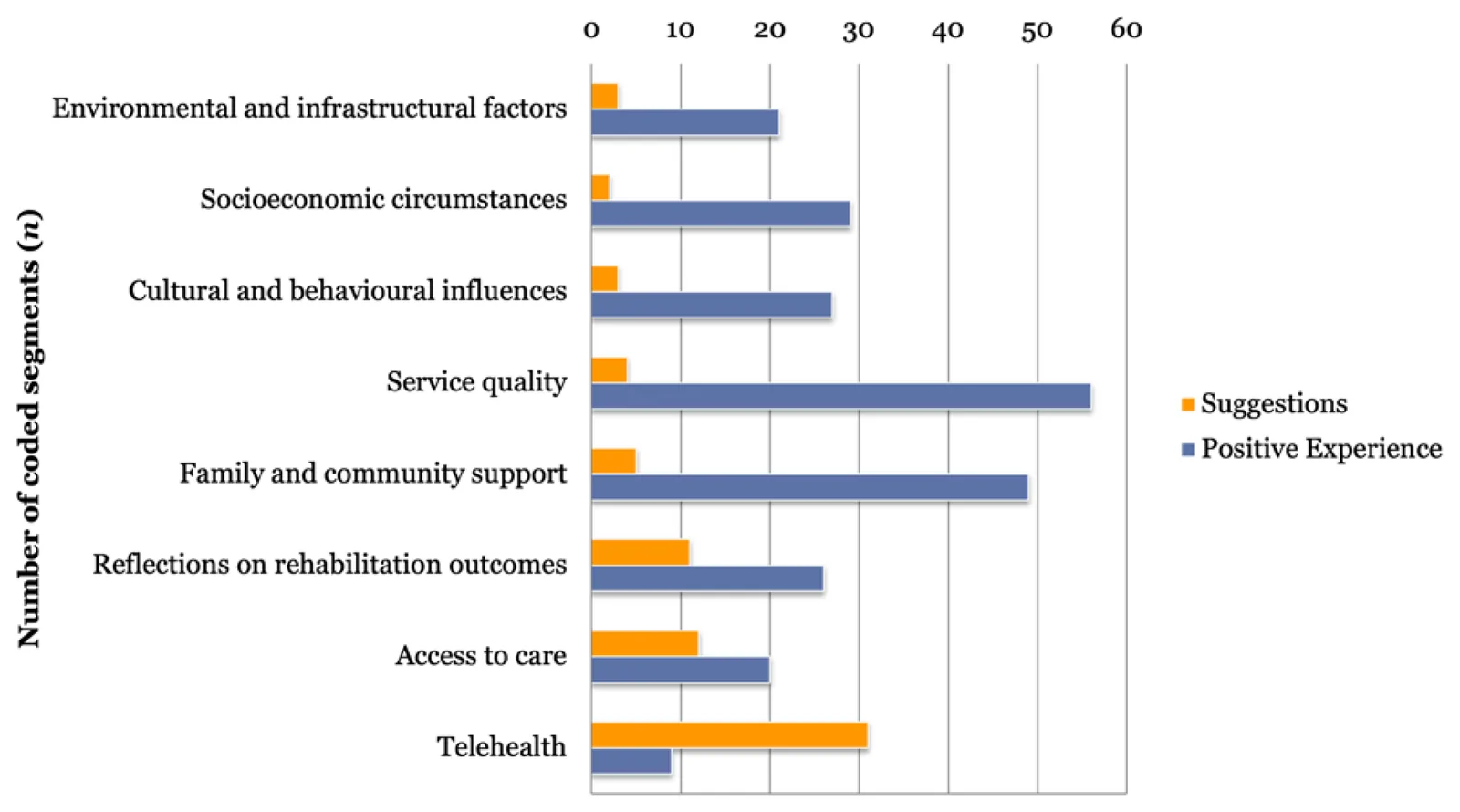
Facilitator types by theme.

**Table 1:** Participant demographics (*N* = 30).

Variable	*n* (%)
**Age group**	

60 to 69	12 (40.0)
70 to 79	11 (36.7)
80 to 89	4 (13.3)
≥ 90	3 (10.0)

**Sex**	

Male	11 (36.7)
Female	19 (63.3)

**Ethnicity**	

Malay	13 (43.3)
Chinese	15 (50.0)
Indian	2 (6.7)

**Diagnosis (grouped)**	

Neurological (e.g., stroke)	18 (60.0)
Functional decline (e.g., deconditioning)	11 (36.7)
Musculoskeletal (e.g., spondylosis)	1 (3.3)

*n* = number of participants; % = percentage of the total sample.

**Table 2:** Summary of thematic analysis: barriers, facilitators and sentiment scores.

Theme	Total count^a^	Barriers (%)^b^	Facilitators (%)^b^	Mean sentiment score^c^
Access to care	113	71.7	28.3	−0.36
Service quality	145	58.6	41.4	−0.06
Socioeconomic circumstances	86	64.0	36.0	−0.21
Cultural and behavioural influences	69	56.5	43.5	−0.03
Environmental and infrastructural factors	64	64.1	35.9	−0.15
Reflections on rehabilitation outcomes	77	51.9	48.1	−0.02
Family and community support	74	27.0	73.0	0.33
Telehealth	67	40.3	59.7	0.25

a Number of coded text segments assigned to the theme;

b Proportion of the coded segments of that theme expressed as barriers or facilitators (sum = 100%);

c Mean sentiment polarity score (range −1 to +1, negative values indicate more negative sentiment).

## Data Availability

All recorded data and transcriptions will be deleted after the retention period specified in the participant consent agreement, in accordance with institutional ethics and data protection requirements. Owing to the confidential and sensitive nature of the qualitative data, the datasets generated and analysed during the current study are not publicly available but may be made available by the corresponding author upon reasonable request, subject to ethical approval.
